# Urbanization increases left-bias in line-bisection: an expression of elevated levels of intrinsic alertness?

**DOI:** 10.3389/fpsyg.2014.01127

**Published:** 2014-10-09

**Authors:** Karina J. Linnell, Serge Caparos, Jules Davidoff

**Affiliations:** ^1^Psychology, Goldsmiths University of LondonLondon, UK; ^2^Psychologie, Université de NîmesNîmes, France

**Keywords:** attention, engagement, control, alertness, spatial attention, spatial bias, laterality, urbanization

## Abstract

Urbanization impairs attentional selection and increases distraction from task-irrelevant contextual information, consistent with a reduction in attentional engagement with the task in hand. Previously, we proposed an attentional-state account of these findings, suggesting that urbanization increases intrinsic alertness and with it exploration of the wider environment at the cost of engagement with the task in hand. Here, we compare urbanized people with a remote people on a line-bisection paradigm. We show that urbanized people have a left spatial bias where remote people have no significant bias. These findings are consistent with the alertness account and provide the first test of why remote peoples have such an extraordinary capacity to concentrate.

## Introduction

Remote peoples living in natural environments have an extraordinary capacity for attentional selection of task-relevant material. They show substantially reduced interference from task-irrelevant information, even to the point where a highly salient distractor (such as a motion singleton) exerts no significant interference (de Fockert et al., 2011). Furthermore, their capacity for selection exceeds that of urbanized controls even when distracting information is more salient to them than to controls: thus, despite their having a perceptual bias to process local information, they show less interference from distracting local information when selecting global information than urbanized controls (as well as less interference from distracting global information when selecting local information; Caparos et al., 2013). In sum, remote peoples are better able to focus their attention on the task in hand. What is more, they are even able to focus on easy tasks (of low perceptual load; Linnell et al., 2013) where attentional engagement is supposed to be limited (Kahneman, 1973). Here we set out to provide the first test of why remote peoples should have such an extraordinary capacity to concentrate.

**Figure 1:**
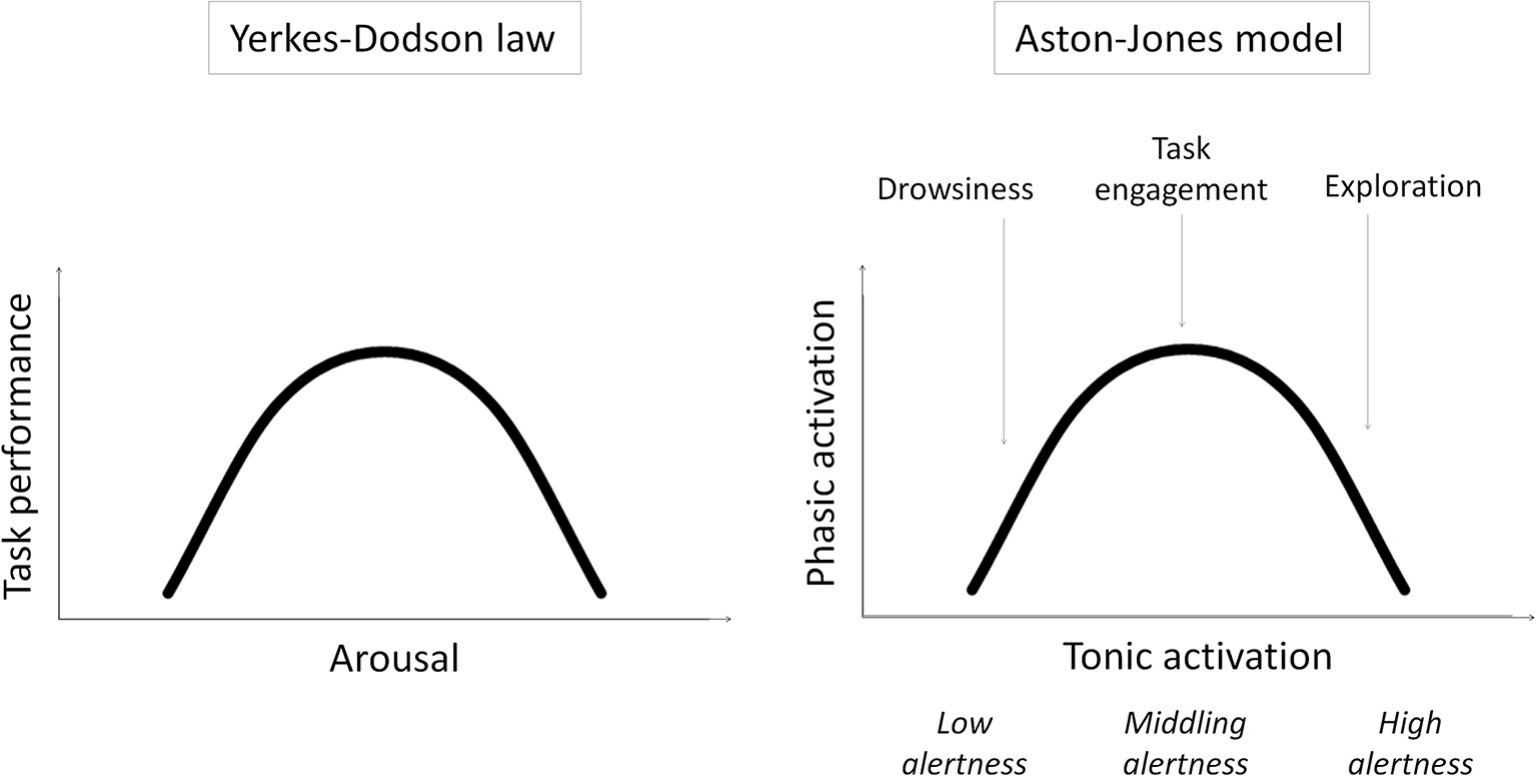
**Schematic illustrations of the Yerkes–Dodson law and the Aston-Jones model and the parallels between them**.

Specifically, we examine our previous speculation that remote peoples have middling levels of intrinsic alertness^1^, optimally suited to task engagement and the selective processing of task-relevant stimuli, while urbanization increases intrinsic alertness, and with it exploration and the processing of task-irrelevant contextual stimuli (Linnell et al., 2013). This speculation is based on the model of Aston-Jones and colleagues (e.g., Aston-Jones et al., 1999; Aston-Jones and Cohen, 2005) and related work (for a review, see Singh-Curry and Husain, 2009) suggesting that exploration and task engagement represent different ways of interacting with the world, distinguished only by different underlying levels of intrinsic alertness. According to the model, intrinsic alertness is expressed by tonic activity in the locus coeruleus-norepinephrine system (LC-NE). Whereas low tonic activity in the LC-NE system—low alertness—is linked to low sensitivity to external stimuli and leads to drowsiness, high tonic activity—high alertness—is linked to high sensitivity to external stimulation and leads to exploration. A middling level of tonic activity—middling alertness—leads to task engagement by enabling selective or phasic activity in the LC-NE system that is time-locked to the presentation of task-relevant stimuli. The model shows that this phasic activity^2^ is reduced if tonic activity is either too low or too high—in states of low or high intrinsic alertness, respectively. In other words, the model results in task engagement following an “inverted-U” function of intrinsic alertness. This is, in essence, a restatement of the Yerkes–Dodson law (Yerkes and Dodson, 1908) where task performance (as driven by task engagement) first improves and then falls off with increasing alertness/arousal (see Figure 1).

Can the extraordinary capacity of remote peoples to engage with the task in hand be explained by this variant of the Yerkes–Dodson law? According to the model, task engagement predominates when intrinsic alertness is at middling levels, and exploration predominates when it is at high levels. The dynamic range of the model is obviously critical to accommodating both resting and stress conditions; however, the proposal which we set out to test here is that urbanization induces, probably through stress-related effects (Lederbogen et al., 2011), elevated levels of intrinsic alertness even under resting conditions and thus shifts the balance in favor of exploration and away from task engagement. This proposal is plausible given the studies showing that stress increases tonic activation in the LC-NE system (for a review, see Aston-Jones et al., 2007).

In order to test the intrinsic-alertness hypothesis, we rely on the right lateralisation of the system that mediates alertness (e.g., Sturm et al., 1999; He et al., 2007; Corbetta and Shulman, 2011). We invoke findings showing that, because of this lateralisation, changes in intrinsic alertness affect relative hemispheric activation patterns and, as a result, left-right spatial biases (Newman et al., 2013). Specifically, decreasing intrinsic alertness with time-on-task results in rightwards moving spatial biases (Manly et al., 2005). Equally, decreases in intrinsic alertness due to sleep deprivation and/or disruption of the circadian rhythm, as well as approaching the nadir of the circadian rhythm, or being on the point of falling asleep, also result in rightwards moving spatial biases (Manly et al., 2005; Schmitz et al., 2011; Bareham et al., 2014). Conversely, interventions that increase intrinsic alertness, whether by phasic alerting or the administration of stimulants, result in leftward moving spatial biases (e.g., Robertson et al., 1998; Sheppard et al., 1999).

Here we reason that if urbanization increases intrinsic alertness it should also shift spatial biases leftward. We compared spatial biases in urbanized and remote groups using the same behavioral paradigm employed in most of the studies reviewed above. Specifically, we used the line-bisection paradigm in a variant called the Landmark test (Milner et al., 1992; Manly et al., 2005) that has been advocated as the most sensitive measure of spatial bias (Jewell and McCourt, 2000). In this paradigm, the subjective midpoint of horizontal lines is measured by presenting transected lines and asking participants to indicate whether the part of the line left or right of the transector appears longer; the direction of any deviation of the perceived midpoint from veridical center reflects greater activation in the contralateral hemisphere, the more so the greater the extent of the deviation (Newman et al., 2013).

The subjectively judged midpoint or point of subjective equality (PSE) measured with this paradigm generally falls left of center, even in high-functioning (urbanized) participants tested under optimal conditions. This phenomenon is known as pseudo-neglect to contrast it with the well-known and very extensive rightward bias in neglect patients with right-hemisphere lesions (which notably ameliorates with phasic alerting; Robertson et al., 1998). Though pseudo-neglect is a rather surprising phenomenon, it is widely accepted as the norm, albeit an anomalous one, and its origin has been little researched (Jewell and McCourt, 2000). If we are correct in our suggestion that urbanized peoples have elevated levels of intrinsic alertness that are too high to promote optimal task engagement, whereas remote peoples have more middling intrinsic alertness, then remote peoples should bisect rightwards of urbanized peoples and show reduced and possibly even absent pseudo-neglect.

## Methods

We measured left-right perceptual bias using the Landmark version of the line-bisection task (Milner et al., 1992; Manly et al., 2005) advocated by Jewell and McCourt (2000).

### Participants

The urban participants were British students from London, UK. The remote participants were Himba individuals, living in traditional villages in the open savannah of north-west Namibia. All Himba participants were monolingual (in Otjiherero) and had had little contact with the Western world; on average, they had been to Opuwo (the only town in the region) only 2.5 times (s.e.m. = 0.4) in their lifetime. To our knowledge, none of the Himba had ever been involved in experimental research before.

Fifty-six British (38 females, mean age = 25.6 years, age range = 17–43 years) and 56 Himba participants (33 females, mean age = 25.4 years, age range = 17–42 years), matched in terms of (1) time of testing and (2) age took part in the experiment. Testing took place between 10.03 am and 4.04 pm local time (mean time = 12.48 pm) with the British participants, and between 10.01 am and 3.58 pm local time (mean time = 12.44 pm) with the Himba participants.

Participants were paid or rewarded in kind (with flour and sugar).

### Stimuli

Stimuli were modeled on those used in Manly et al. (2005) and were presented using E-Prime 1.0 (Schneider et al., 2002) on a 20-in CRT monitor (SONY Trinitron F520) at a viewing distance of 70 cm.

Stimuli consisted of transected lines, the target stimuli, each followed by a mask. Both target stimuli and the mask were presented in black on a white background along the horizontal midline of the screen.

**Figure 2:**
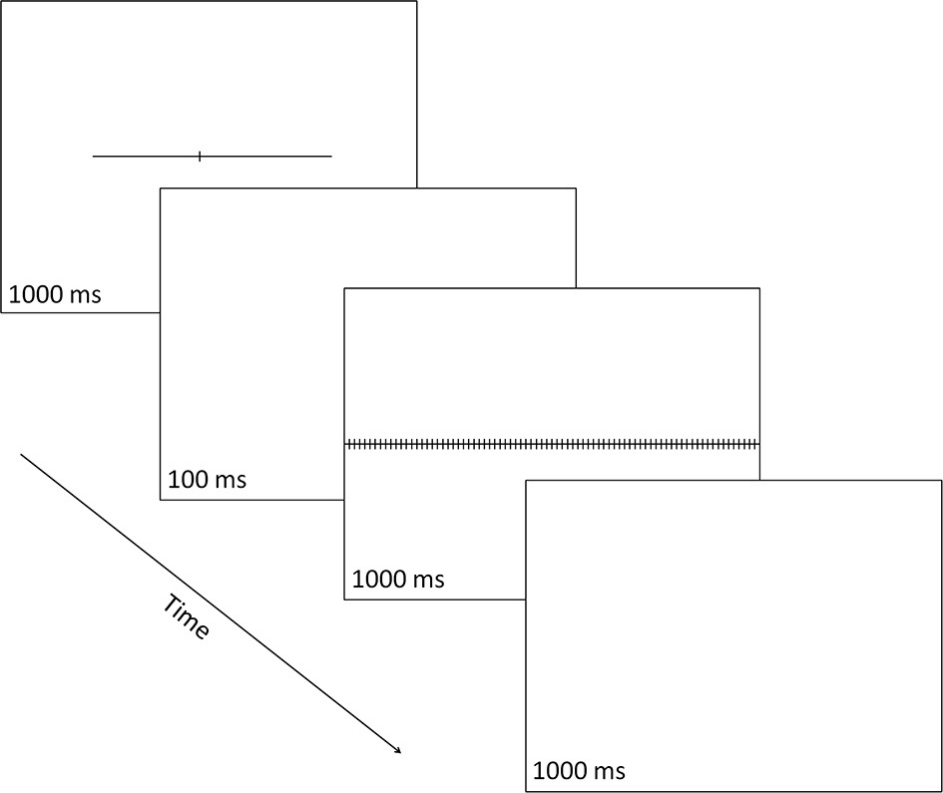
**A schematic illustration of a single trial in which the line transector fell to the left of line center and the correct response was a right button-press signaling that the part of the line that was longer was the right one**. Numbers indicate exposure durations in milliseconds (ms).

Each target stimulus was a horizontal line subtending 18.8° (or degrees of visual angle) in length and 0.1° in width, and transected by a small vertical line subtending 0.8° in length and 0.1° in width (see Figure 2). The vertical line could transect the horizontal line at one of seven possible locations, thus creating seven different target stimuli defined by the length of the horizontal-line parts left and right of the transector. Three targets had a left part longer than the right part (i.e., 10.4° vs. 8.4°, 10.0° vs. 8.8°, or 9.6° vs. 9.2°, respectively), one target had left and right parts equal in length (i.e., 9.4° vs. 9.4°) and three targets had a left part shorter than the right part (i.e., 9.2° vs. 9.6°, 8.8° vs. 10.0°, or 8.4° vs. 10.4°, respectively). Each of the seven types of target stimuli was equally often presented centered at the vertical midline of the display, jittered 1.1° to the left, or jittered 1.1° to the right, creating 21 distinct target displays.

The mask was a horizontal line that stretched from the left to the right edge of the CRT along the horizontal midline. It was crossed by 86 equidistant vertical lines, each subtending 0.8° in length and 0.1° in width and separated from each other by 0.4°.

### Procedure

On each trial, the target stimulus, the transected line, was shown for 1000 ms and was followed by a 100-ms blank; then the mask was shown for 1000 ms, followed by a 1000-ms blank, after which the next trial started (even if the participant has not made any response). Responses could be made at any point during each trial after the onset of the transected line. The participant made a two-alternative forced-choice (2-AFC) response to indicate which part of the line (left or right) was longer: using a two-button response box, s/he pressed the left button (with their left hand) to indicate a “left” choice or the right button (with their right hand) to indicate a “right” choice.

Before starting the test trials, participants completed a 10-trial practice session in which the left and right parts of the target were distinctly different (i.e., they measured, respectively, 10.8° and 8.0°, or 8.0° and 10.8°). During the subsequent test phase, each of the 21 distinct target displays described above was presented four times, in randomized order.

Testing with the Himba participants took place on the outskirts of traditional Himba villages, inside a large testing tent placed in a shaded area. Testing with the British participants took place inside a quiet and moderately lit testing room in London, UK. For the Himba, instructions were given with the help of an interpreter who was naive to the purpose of the study.

## Results

**Figure 3:**
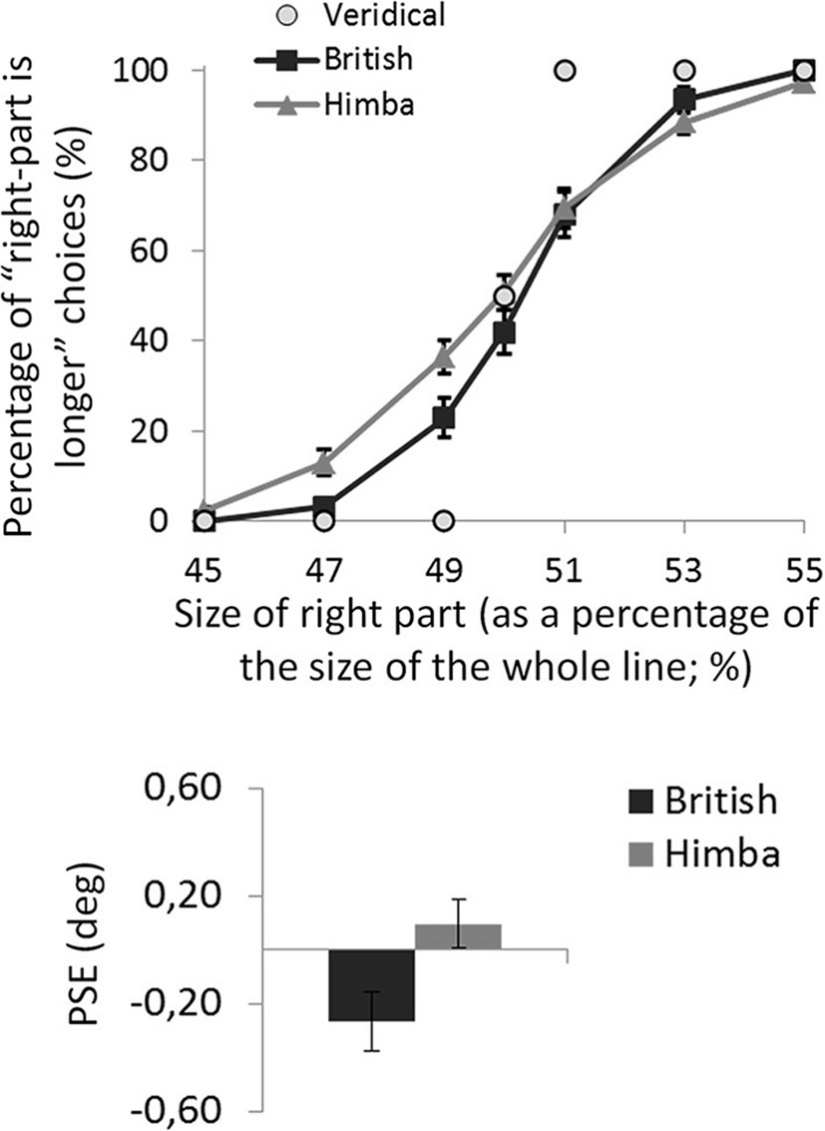
**A graph comparing the psychometric functions and group mean line-bisection PSEs for urbanized (British) and remote (Himba) groups**. Error bars depict ±1 s.e.m.

For each participant, the mean percentage of “right part is longer” responses was calculated for the seven target lines defined by the position of the transector relative to the line. The task was well performed: all participants produced accuracies in excess of 90% for the most extreme line bisections in the test phase, showing that they understood the task (see Figure 3 illustrating the group psychometric functions).

For each participant, the PSE was computed (in degrees of visual angle), defining their threshold for deciding that the right side of the transected line was longer than the left side ^3^. Negative PSEs indicated that participants perceived the middle of the line to fall to the left of veridical center (i.e., consistent with the “pseudoneglect” pattern of bisection typical of healthy urbanized participants). In contrast, positive PSEs indicated that participants perceived the middle of the line to fall to the right of veridical center (i.e., consistent with the “neglect” pattern of bisection).

An independent-samples *t*-test showed that British participants had significantly lower PSEs than Himba participants, *t*_(110)_ = 2.2, *p* = 0.033, *d* = 0.410 (see Figure 3). While British participants had PSEs significantly lower than zero (i.e., they bisected significantly to the left and thus displayed pseudo-neglect), *t*_(55)_ = 2.7, *p* = 0.010, *d* = 0.497, the PSE of Himba participants was not significantly different from zero or veridical center, *t*_(55)_ = 0.7, *p* = 0.476, *d* = 0.136. Indeed, these PSE-based findings are supported by focusing on performance with just the perfectly bisected lines (Manly et al., 2005). The Himba participants judged the right side of perfectly bisected lines to be longer than the left 51% of the time in contrast to the British participants who judged the right side to be longer 42% of the time.

**Figure 4:**
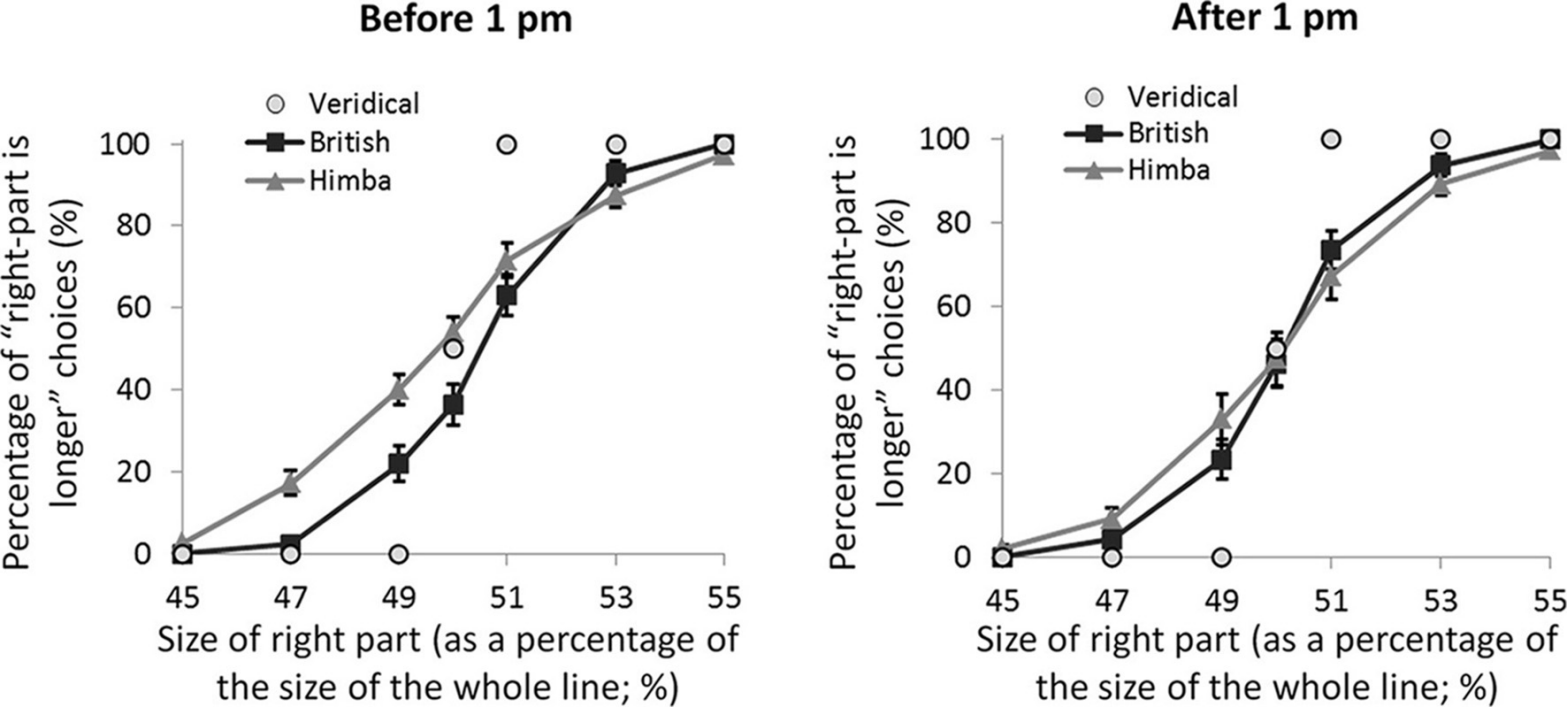
**A graph comparing the psychometric functions for urbanized (British) and remote (Himba) groups, both before 1 pm and after 1 pm**. Error bars depict ±1 s.e.m.

If these spatial biases are really due to alertness differences, they ought to be affected by time of testing. Almost exactly half our participants were tested before 1 pm local-time and almost exactly half after 1 pm (with Himba and British participants pair matched for exact time of testing). Figure 4 shows the psychometric functions for both groups split by time of testing (namely, before and after 1 pm). For the PSEs, there was a significant interaction between group and time of testing, *F*_(1, 108)_ = 4.2, *p* = 0.042, η^2^_*p*_ = 0.038, such that there was a significant group difference earlier in the day, *t*_(55)_ = 2.7, *p* = 0.009, *d* = 0.697, that vanished later, *t*_(55)_ = 0.1, *p* = 0.917, *d* = 0.040^4^. The group difference early in the day was founded on the fact that, while British participants showed a significant leftward bias (*M* = −0.42, s.e.m. = 0.16), *t*_(28)_ = 2.5, *p* = 0.017, *d* = 0.660, Himba participants did not show a significant bias (*M* = 0.28, s.e.m. = 0.17), *t*_(26)_ = 1.4, *p* = 0.174, *d* = 0.360. Later in the day, neither British participants (*M* = −0.10, s.e.m. = 0.17) nor Himba participants (*M* = −0.08, s.e.m. = 0.16) showed a significant bias, respectively, *t*_(28)_ = 1.0, *p* = 0.322, *d* = 0.255, and *t*_(28)_ = 0.4, *p* = 0.666, *d* = 0.104. Figure 4 shows that the PSE-based findings are again supported by performance with just the perfectly bisected lines.

## General discussion

Performance on the Landmark version of the line-bisection task (Milner et al., 1992; Manly et al., 2005) differed significantly between remote (traditional Himba) and urbanized (British) participants with similar age and gender spread and tested at matched times of day. While overall the urbanized group bisected significantly to the left of center, replicating the standard pseudo-neglect phenomenon (Jewell and McCourt, 2000), the remote group produced bisections that were, if anything, right of veridical center although they did not differ significantly from veridical performance (see Figure 3).

While our findings are consistent with remote peoples having less activity in the right-lateralised system mediating alertness—that is lower levels of intrinsic alertness—than urbanized groups, we must also consider alternative or additional explanations for our findings. Although alertness does indeed influence line-bisection performance in systematic ways (e.g., Manly et al., 2005), there are other factors that are known to influence it which we must consider, notably age, time of day, and reading direction (see Jewell and McCourt, 2000, for a review). While we controlled for the first two, we could not control for the last: the traditional Himba that we tested were all illiterate, whereas our British controls all read from left to right. Despite the fact that the research on reading direction is inconclusive (e.g., Chokron et al., 1998; Nicholls and Roberts, 2002), experience with left-to-right scanning in our British participants could have produced a slight leftwards bias absent in the Himba. We argue, however, that this possibility is inconsistent with the fact that the Himba and British spatial biases were indistinguishable in the latter part of the day (see Figure 4). More generally, the sensitivity of the group difference in spatial bias to time of day is difficult to explain in terms of other factors that may vary between groups and that are lateralized (and that could in theory produce group differences in spatial bias) but that should not vary with time of day, except of course if they are driven by alertness differences (e.g., global-local bias; Caparos et al., 2012). Similarly, the time-of-day effects seem to rule out the possibility that it is attentional engagement itself that explains the group differences in bisection (such that increased engagement in remote groups produces more veridical bisections) since there is no reason to suppose that engagement should vary with time of day except if, as we propose here, it is driven by alertness. While we cannot completely rule out the influence of other factors, it is most parsimonious to argue that the pattern of spatial biases that we report here originates in group differences in overall intrinsic alertness which are linked to different patterns of diurnal variation in alertness (e.g., Thayer, 1989).

In future work comparing remote and urbanized groups, it will be important to collect more direct markers of intrinsic alertness and attentional engagement, and of the balance between tonic vs. phasic activity in the locus coeruleus-norepinehrine (LC-NE) system. A behavioral proxy for activity in the LC-NE system which is amenable to field testing is pupil dilation (Gilzenrat et al., 2010). For example, Smallwood and colleagues (e.g., Smallwood et al., 2011) have shown that increasing task difficulty decreases baseline pupil dilation and increases task-related pupil dilation. This is consistent with increasing task difficulty motivating a decrease in tonic LC-NE activity/alertness and an increase in phasic LC-NE activity/task engagement. Combining the present finding that remote groups show reduced leftward spatial biases with previous findings that they can engage attention even on easy tasks of low load (Linnell et al., 2013), leads us to the prediction that—in easy as in hard tasks—their baseline pupil dilation will be low and their task-related increases in pupil dilation high.

Pending such direct evidence, we interpret our present findings as being consistent with remote groups having lower intrinsic-alertness levels than urbanized groups. Thus, the greater task engagement and immunity to distraction of remote peoples (de Fockert et al., 2011; Caparos et al., 2013; Linnell et al., 2013) seems to be associated with decreases in intrinsic alertness. This interpretation allows us to discount the very important possibility that the previously reported increased engagement of remote peoples was an artifact of the novelty of the testing situation (whether arising from the use of luminous displays and other electronic gadgetry or the different ethnicity and lifestyle of the experimenters). Novelty is arousing and should increase alertness not decrease it (e.g., Yanaka et al., 2010). Rather, the greater task engagement and immunity to distraction of remote peoples seems to be associated with levels of intrinsic alertness that are lower than those in urbanized groups. As outlined in the introduction to this manuscript, this is compatible with previous suggestions that attentional engagement is optimal at middling levels of intrinsic alertness and falls off with higher levels (Yerkes and Dodson, 1908; Aston-Jones and Cohen, 2005; Singh-Curry and Husain, 2009). It opens the door to the possibility that the standard leftward-bisection bias in the healthy (urbanized) populations reported in the literature is not a reflection of a “natural” state but rather of a hyper-vigilant one (albeit not as extreme, for example, as that in powerless urbanized people; Wilkinson et al., 2010). In this context, it is noteworthy that leftward biases in non-remote groups have only been reported to arise in early adolescence; while it has been speculated that the late expression of the leftwards bias is an artifact of the delayed development of the corpus callosum (Hausmann et al., 2003), its timing could be linked to the age at which urbanization first results in elevated levels of intrinsic alertness/hemispheric imbalances. It is also noteworthy that participants who are high in mindfulness, and who like the remote group presented here exhibit increased attentional control (e.g., Jha et al., 2007), also exhibit more balanced hemisphere-activation patterns (Aftanas and Golosheykin, 2005).

If the pseudo-neglect and hemispheric asymmetry in urbanized groups does reflect elevated intrinsic alertness, the next question we need to answer is whether the increase in alertness is reversible. It is tempting to conclude that it is and to speculate that the restorative effects of short-term exposure to the natural environment on cognitive function (Kaplan, 1995; Berman et al., 2008) are also linked to reductions in intrinsic-alertness levels. However, this and other analogies between short-term reversible effects and the effects reported here must be treated with extreme caution given that urban upbringing has been reported to impact different aspects of the alerting network to more short-term urban living (Lederbogen et al., 2011). Thus, it is possible that urban exposure exerts both non-reversible and reversible effects and that these effects are underpinned by different mechanisms.

In sum, our findings are consistent with remote groups having reduced intrinsic-alertness levels compared to urbanized ones. The dynamic and unpredictable nature of the urban environment may make increased alertness and exploration more adaptive for urban groups but this may come at the cost of reduced immersion in the “here and now” of the task in hand. In contrast, remote groups appear to be able to engage their attention even on easy tasks (low in perceptual load; Linnell et al., 2013) that have previously been thought to be incapable of engaging us (Kahneman, 1973) and to remain undistracted even by the most salient (movement-singleton) distractors (de Fockert et al., 2011) that have previously been reported always to distract us (Theeuwes, 2010). Remote groups may show us that optimizing intrinsic-alertness levels for task engagement (Yerkes and Dodson, 1908; Aston-Jones and Cohen, 2005) can support attentional performance that outstrips the limits of what has heretofore been thought possible.

### Conflict of interest statement

The authors declare that the research was conducted in the absence of any commercial or financial relationships that could be construed as a potential conflict of interest.

## Footnotes

^1^Here we use intrinsic alertness to signify internally controlled wakefulness or arousal.

^2^Phasic activity here can be equated with extrinsic alertness, insofar as extrinsic alertness is defined as the phasic response to external stimuli (driven by tonic activity/internally controlled wakefulness or arousal), as opposed to externally controlled wakefulness or arousal (driven, for example, by alerting cues).

^3^We fitted the data for each participant with the model: *p* = φ([*k – d*]/σ), where *p* is the probability of choosing the larger part of the target, φ(*z*) is the inverse cumulative distribution function for a standard normal distribution, *k* is the required threshold for deciding that the larger part of the target was the larger one, *d* is the length difference between the two parts of the target (in degrees of visual angle) and σ is the standard deviation of the normally distributed noise from all sources.

^4^Time of testing effects were not significant for either group.
